# Design and Optimization of 3D-Printed Variable Cross-Section I-Beams Reinforced with Continuous and Short Fibers

**DOI:** 10.3390/polym16050684

**Published:** 2024-03-02

**Authors:** Xin Zhang, Peijie Sun, Yu Zhang, Fei Wang, Yun Tu, Yunsheng Ma, Chun Zhang

**Affiliations:** 1 School of Aeronautics, Northwestern Polytechnical University, Xi’an 710072, China; zhangxin504@mail.nwpu.edu.cn; 2 Institute of Aircraft Composite Structures, Northwestern Polytechnical University, Xi’an 710072, China; 3 School of Mechanics, Civil Engineering and Architecture, Northwestern Polytechnical University, Xi’an 710072, China; 4 School of Aircraft, Xi’an Aeronautical Institute, Xi’an 710077, China; 5 Key Laboratory of Pressure Systems and Safety, School of Mechanical and Power Engineering, Ministry of Education, East China University of Science and Technology, Shanghai 200237, China; 6 Shandong Chambroad Holding Group Co., Ltd., Binzhou 256599, China

**Keywords:** 3D printing, optimization, continuous carbon fiber, I-beam, equal strength

## Abstract

By integrating fiber-reinforced composites (FRCs) with Three-dimensional (3D) printing, the flexibility of lightweight structures was promoted while eliminating the mold’s limitations. The design of the I-beam configuration was performed according to the equal-strength philosophy. Then, a multi-objective optimization analysis was conducted based on the NSGA-II algorithm. 3D printing was utilized to fabricate I-beams in three kinds of configurations and seven distinct materials. The flexural properties of the primitive (P-type), the designed (D-type), and the optimized (O-type) configurations were verified via three-point bending testing at a speed of 2 mm/min. Further, by combining different reinforcements, including continuous carbon fibers (CCFs), short carbon fibers (SCFs), and short glass fibers (SGFs) and distinct matrices, including polyamides (PAs), and polylactides (PLAs), the 3D-printed I-beams were studied experimentally. The results indicate that designed and optimized I-beams exhibit a 14.46% and 30.05% increase in the stiffness-to-mass ratio and a 7.83% and 40.59% increment in the load-to-mass ratio, respectively. The CCFs and SCFs result in an outstanding accretion in the flexural properties of 3D-printed I-beams, while the accretion is 2926% and 1070% in the stiffness-to-mass ratio and 656.7% and 344.4% in the load-to-mass ratio, respectively. For the matrix, PAs are a superior choice compared to PLAs for enhancing the positive impact of reinforcements.

## 1. Introduction

Fiber-reinforced composites (FRCs) are replacing metal materials and are emerging as the primary structural material for lightweight transportation and aviation, such as drones, new energy vehicles, and high-speed rails [1,2]. Due to their exceptional strength, stiffness, fatigue resistance, and corrosion resistance, the applications of FRCs in structures are steadily growing, especially in main load-bearing structures [3,4]. For example, the utilization of pultruded carbon fiber-reinforced beams on large wind turbine blades has become a consensus in the energy industry [5]. Molds, however, have historically restricted the design and production of conventional FRCs, resulting in high costs and drawn-out production cycles [6]. Three-dimensional (3D) printing, also known as additive manufacturing, usually refers to the process of freely depositing patterns in the XY plane and stacking them layer by layer in the z direction to create a structure [7,8]. The emergence of this technology greatly reduces material waste in the manufacturing process and makes rapid manufacturing possible [9]. Moreover, it is a promising technology for manufacturing composite materials, which has the potential to free FRCs from the limitations of molds [10,11]. When producing porous structures using conventional composite materials, it is typically required to utilize compound molds. By combining 3D printing with FRCs, it is feasible to achieve quick one-shot manufacturing without the use of molds [9].

In recent years, 3D printing of composite materials based on fused deposition modeling (FDM) has been developed due to the affordable equipment and consumables [12,13]. The addition of fibrous reinforcements, such as continuous and short fibers, has demonstrated significant enhancements in mechanical properties of bending-resistant structures [14,15,16]. By employing continuous carbon fibers (CCFs), the tensile and bending strengths of 3D-printed structures can be increased from 28 MPa and 53 MPa to 80 MPa and 59 MPa, respectively, compared to the structures made of polylactides (PLAs) [14]. Moreover, if a sizing agent is used for pretreatment, the tensile and flexural strengths will be increased by 3.25 and 2.94 times [14], respectively. Adding short carbon fibers (SCFs) to the 3D-printed polycarbonate structure has resulted in a 127% increase in the modulus and an 18.9% increase in the flexural strength [15]. Furthermore, when 15% and 30% of SGFs were added to the acrylonitrile butadiene styrene (ABS) matrix, the flexural properties enhanced by 44% and 59%, respectively [16].

With the introduction of 3D printing, the designability of CCFs has been significantly improved due to the removal of mold restrictions. Multiple types of beams with diverse shapes were fabricated using 3D printing and were subsequently evaluated by mechanical investigation [17,18,19,20,21]. Distinct infill patterns, including rhombus, square, and classic hexagonal, were utilized to manufacture 3D-printed cellular-cored sandwich beams made of CCF composite materials [17]. The three-point bending experimental results indicate that the flexural modulus and bending strength of these beams are comparable with those of competing structures made of aluminum or glass fiber-reinforced composites. The 3D-printed PLA-based T-beam exhibits a flexural strength of 85 MPa and a flexural modulus of 3.5 GPa with an 8 wt.% of SCFs, while the flexural strength increased to 255 MPa and the flexural modulus could reach 16 GPa with an 8 wt.% of CCFs [18].

Despite the significance of I-beams in several industries such as machining [19], architecture [20], and energy [21], there is currently an absence of research on 3D-printed fibrous I-beams. The pultrusion process is currently the most efficient method for manufacturing composite I-beams. However, it is not capable of producing structures with variable cross-sections [22]. Advancements in 3D-printing technology enable the flexible design and manufacturing of I-beams with customizable cross-sections. Nevertheless, the existing research regarding I-beam structural design and the optimization of 3D-printed fiber-reinforced composite materials remains restricted, as well as material selection.

In the present work, the primary objective is to enhance the flexural properties of 3D-printed I-beams through structural design and optimization. The 3D printing of I-beams was conducted with three unique configurations and seven diverse materials. Three-point bending testing was performed to validate the flexural properties of various configurations, reinforcements, and matrix materials. Beams are typical and vital components that withstand bending loads and are extensively utilized in the manufacturing, building, energy, and other industries. This study aims to offer a reference for the design, optimization, and material selection and to guide the utilization of 3D-printed I-beams in lightweight, bending-resistant structures, which might improve the implementation of 3D-printed composite beam structures in commercial applications.

## 2. Materials and Methods

### 2.1. 3D-Printing Process

**Figure 1:**
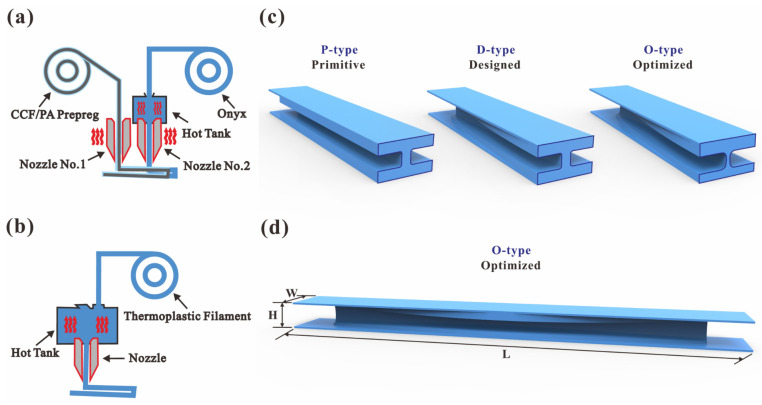
Schematic diagrams of the 3D-printing process and structural configurations: (**a**) a scheme of the Mark Two desktop 3D printer, (**b**) a scheme of the HB250 desktop 3D printer, (**c**) the three configurations of 3D-printed I-beams, and (**d**) the geometry parameters of the 3D-printed I-beams.

Two types of 3D printers were utilized to fabricate continuous and chopped fiber-reinforced composite I-beams. The scheme of the Mark Two desktop 3D printer (from Markforged Inc., Waltham, MA, USA) is illustrated in Figure 1a. There are two printing nozzles in the Mark Two 3D printer, one for printing continuous carbon fiber (CCF)-reinforced polyamide prepregs and the other for printing Onyx filaments. The adoption of CCF/PA prepregs could enhance the mechanical properties of the structures, whereas the Onyx materials are applied to smooth surfaces and fill corners to ensure the form accuracy of parts. The CCF/PA prepregs and Onyx filaments were both purchased from Markforged Inc., Waltham, MA, USA.

Figure 1b shows an illustration of the HB250 desktop 3D printer from Dongguan Xianglong 3D Technology Co., Ltd., Dongguan, China. The nozzle extruded the thermoplastic filament after it melted inside the hot tank. It is capable of printing a range of thermoplastic materials that have extrusion temperatures below 350 degrees, including PAs and PLAs with short carbon fibers (SCFs) or short glass fibers (SGFs). The grades and basic mechanical properties of filaments used in 3D printing are summarized in Table 1, whereas the printing parameters are listed in Table 2. The filaments with discontinuous or no reinforcements were both purchased from the eSUN 3D printing department, Shenzhen Guanghua Weiye Co., Ltd., Shenzhen, China.

Carbon fibers and carbon fiber-reinforced polymers (CFRPs) have had significant advancements in recent years, with a compound annual growth rate (CAGR) of around 12.5% globally over the past two decades [2]. An important reason for this is that carbon fibers offer several benefits, including an exceptional strength-to-weight ratio, a superior stiffness-to-weight ratio, and an excellent resistance to chemicals and heat. In this study, the tensile strength of adopted T300 carbon fibers achieves 3530 MPa, while the tensile modulus could reach up to 230 GPa [2]. These values significantly exceed the tensile strength (2400 MPa) and tensile modulus (72 GPa) of adopted E-glass fibers [23].

It should be noted that three configurations of I-beams were 3D printed, as presented in Figure 1c. In order to clearly illustrate the distinctions between the configurations, the beams are dissected and displayed in a bisected form. The primitive configuration (P-type) has a constant cross-section throughout the entire design interval length (210 mm). The designed configuration (D-type) modifies the dimensions of the I-beam flanges in terms of width and thickness, while maintaining uniformity of the top and bottom flanges. The optimized configuration (O-type) not only adjusts the dimensions of the I-beam flanges in terms of width and thickness of both top and bottom flanges but also varies the thickness of different cross-sections across the whole design interval length. Figure 1d. displays the entire O-type beam with a length (*L*), width (*W*), and height (*H*) of 250 mm, 25 mm, and 15 mm, respectively. The process of obtaining these arrangements will be further elucidated in Section 2.3, Section 2.4 and Section 2.5. Here, I-beams with seven different materials and three different configurations were 3D printed to compare the flexural properties of different configurations, reinforcements, and matrices experimentally.

### 2.2. Three-Point Bending Testing

**Figure 2:**
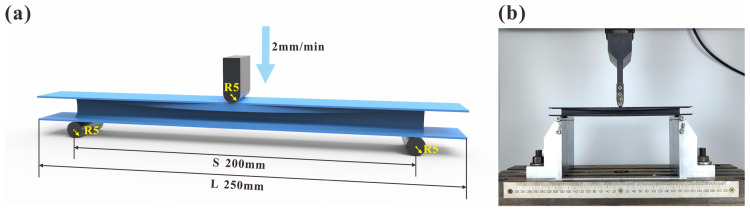
A (**a**) schematic diagram and (**b**) snapshot of the three-point bending testing.

The three-point bending test loading method is considered a conventional experimental technique for bending-performance evaluation due to its straightforwardness and the relative simplicity of the fixture and test parameters [9]. Therefore, in this study, the three-point bending test was adopted instead of the four-point bending test. The DNS200 universal electronic testing machine from the Changchun Research Institute for Mechanical Science Co., Ltd., (Jilin, China) was adopted to perform three-point bending testing at a speed of 2 mm/min. The testing machine automatically collects the experimental load and displacement data. The schematic diagram of the testing is shown in Figure 2a, and the corresponding snapshot is presented in Figure 2b. The radius of the loading head and the supporting roller is 5mm, and the span of the supporting roller is 200 mm.

The load-to-mass ratio (*LMR*) and the stiffness-to-mass ratio (*SMR*) were chosen as indicators to quantify the bending performance of the 3D-printed I-beams. The load-to-mass ratio (*LMR*) was calculated by dividing a maximum bending load by the mass of the beams:

(1) $$LMR=\frac{F_{max}}{m}$$

where *F_max_* is the maximum bending load, while *m* is the mass of the beams.

The stiffness-to-mass ratio (*SMR*) was calculated by dividing the stiffness of the linear stage of the load-to-displacement curve by the mass of the beams:

(2) $$SMR=\frac{E}{m}$$

where *E* is the stiffness of the linear stage, while *m* is the mass of the beams.

### 2.3. Design of Configuration

The primary determinant of strength for beams in engineering is the bending stress in the normal direction. In general, the shear stress strength requirements could also be accommodated when the beam is designed according to the normal stress strength criteria, as follows:

(3) $$\sigma_{max}=\frac{M_{max}}{W_{z}}\leq [\sigma ]$$

where *σ_max_* is the maximum bending stress in the normal direction, *M_max_* is the maximum bending moment, *W_z_* is the flexural modulus of the section, and [*σ*] is the allowable normal stress of the material.

When the beam is subjected to three-point bending loading with a span (*S*) and a load (*F*), as shown in Figure 2a, the bending moment *M* (*x*) on the section is a function of the distance *x* between the section and the nearest supporting roller:

(4) $$M\left(x\right)=\frac{F}{2}x(0\leq x\leq \frac{s}{2})$$

This indicates that the bending moment decreases as the distance from the center increases. According to the equal-strength philosophy [24,25], it is advisable to design a series of suitable cross-sections and to reduce material in areas where the bending moment is relatively low. For the I-beam, the flexural modulus of the section is the moment of inertia relative to the neutral layer (*I*), as follows:

(5) $$I=\frac{t_{w}h_{w}^{3}}{12}+2\times (\frac{bt^{3}}{12}+bt\times (\frac{h_{w}+t}{2}{)}^{2})$$

where *t* is the thickness of the upper and lower beam flanges, *t_w_* is the thickness of the web, *h* is the height of the I-beam, *h_w_* is the height between the upper and lower beam flanges, and *b* is the width of the upper and lower e beam flanges.

Material arrangement can be optimized by designing the width (*b*) and thickness (*t*) of the upper and lower beam flanges and the height between them at different locations. The design of simultaneous variable widths and thicknesses of beam flanges in the form of a hyperbola was adopted in this study, and their specific geometry parameters of configuration are shown in Section 2.5.

### 2.4. Optimization of Configuration

#### 2.4.1. Finite Element Analysis

**Figure 3:**
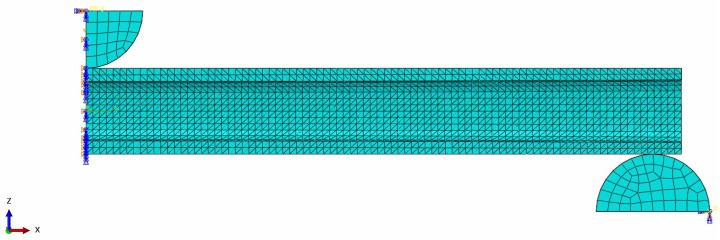
The finite element mode for three-point bending loading conditions.

Catia (version V5-2016) was utilized for modeling and implementing parameterization. Subsequently, the commercial finite element software Abaqus (version 2022) was introduced for a finite element analysis (FEA). Due to the structural symmetry of the 3D-printed beams, a half-beam model is created by establishing symmetry along the positive X-axis direction around the YZ plane, as seen in Figure 3. Consequently, symmetric boundary constraints are then applied to the model. The static analysis step was adopted. A rigid supporting roller and a loading head with a radius of R5 were established. The global size of meshes was 1.2 mm, and the type of meshes was C3D4. The number of meshes was around 4600, while the number of nodes was about 11,000.

Contact constraints were established between the supporting roller, the loading head, and the beam. The tangential friction coefficient was set to 0.2, whereas the normal behavior was defined as hard contact. Fixed boundary conditions (*U*_1_ = *U*_2_ = *U*_3_ = *UR*_1_ = *UR*_2_ = *UR*_3_ = 0) were assigned at the referenced point on the supporting roller, while displacement boundary conditions (*U*_1_ = *U*_2_ = 0, *U*_3_ = 5) were defined at the referenced point on the loading head.

#### 2.4.2. Indexes and Objective Functions

**Figure 4:**
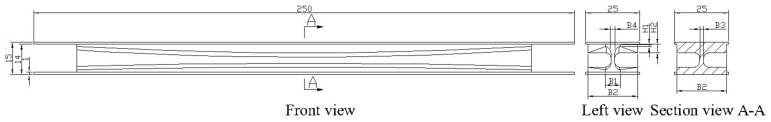
Optimizable parameters of the 3D-printed I-beams.

As shown in Figure 4, six optimizable parameters were constructed for the 3D-printed I-beams, including *B*_1_, *B*_2_, *B*_3_, *H*_1_, *H*_2_, and *R*, and R = *B*_4_/*B*_3_. The aim of structural optimization is to realize the design of variable cross-sections by manipulating these optimizable parameters.

To achieve the variable cross-section design of the 3D-printed I-beams while ensuring their structural integrity and improving their structural efficiency, the following conditions need to be fulfilled: (1) The structure exhibits minimal deflection deformation when subjected to the identical boundary condition, indicating a high level of bending stiffness. (2) To increase the structure’s desirable load-bearing capacity, the stress level under the assigned boundary condition needs to be decreased; here, the first principal stress has been chosen as the index. (3) The mass of the structure ought to be minimized for the highest load-to-mass ratio. Since the density of the same material is identical, the volume can also be utilized to denote structural mass.

The bending stiffness, the first principal stress, and the volume were utilized as indexes to evaluate the 3D-printed I-beams. The constructed objective functions are as follows:

(6) $$\begin{array}{c} \left\{\begin{array}{c} \max f_{1}=W\left(B_{1},\;B_{2},\;B_{3},\;H_{1},H_{2},\;R\right)\\ \max f_{2}=\sigma \left(B_{1},\;B_{2},\;B_{3},\;H_{1},H_{2},\;R\right)\\ \max f_{3}=V\left(B_{1},\;B_{2},\;B_{3},\;H_{1},H_{2},\;R\right) \end{array}\right.\\ s.t.\;\;\;\;0<B_{1}\leq B_{2}\\ \;0<B_{1}\leq 25\\ \;0<RB_{3}+2\leq B_{1}\\ 0<H_{1}\leq 7.5\\ 0<H_{2}\leq 7.5 \end{array}$$

where *W* (*x*_1_, *x*_2_, …, *x*_6_) is the function of the bending stiffness; *σ* (*x*_1_, *x*_2_, …, *x*_6_) is the function of the first principal stress; and *V* (*x*_1_, *x*_2_, …, *x*_6_) is the function of the volume.

Due to the diverse units of the three objective functions, there are significant numerical disparities that impact the outcomes of an algorithmic analysis. Therefore, it is necessary to normalize the functions. The Latin hypercube sampling technique is employed to sample inside the feasible space and to generate a dataset. This dataset is then used to construct a surrogate model, which serves as a substitute for the time-consuming finite element analysis. The mean and variance of the dataset were computed, followed by normalization using the z-score standardization procedure. The final objective function is as follows:

(7) $$f_{i}^{*}=\frac{f_{i}-\mu_{i}}{\sigma_{i}}(i=1,\;2,\;3)$$

where *μ* is the mean of the dataset, and *σ* is the variance of the dataset.

#### 2.4.3. Multi-Objective Optimization Analysis

The simultaneous optimization of a 3D-printed I-beam necessitates the consideration of three objective functions, and the optimal solutions of these functions are contradictory. Hence, it is necessary to coordinate the goal function in order to achieve the Pareto optimal solution within the feasible space. The final multi-objective optimization model for the I-beam design is obtained by incorporating weight values to regulate the influence of the objective function. The final multi-objective is as follows:

(8) $$\min f=(-w_{1}f_{1}^{*},w_{2}f_{2}^{*},w_{3}f_{3}^{*})$$

where *w*_1_ = 1.16, *w*_1_ = 1.2, and *w*_1_ = 1.

**Figure 5:**
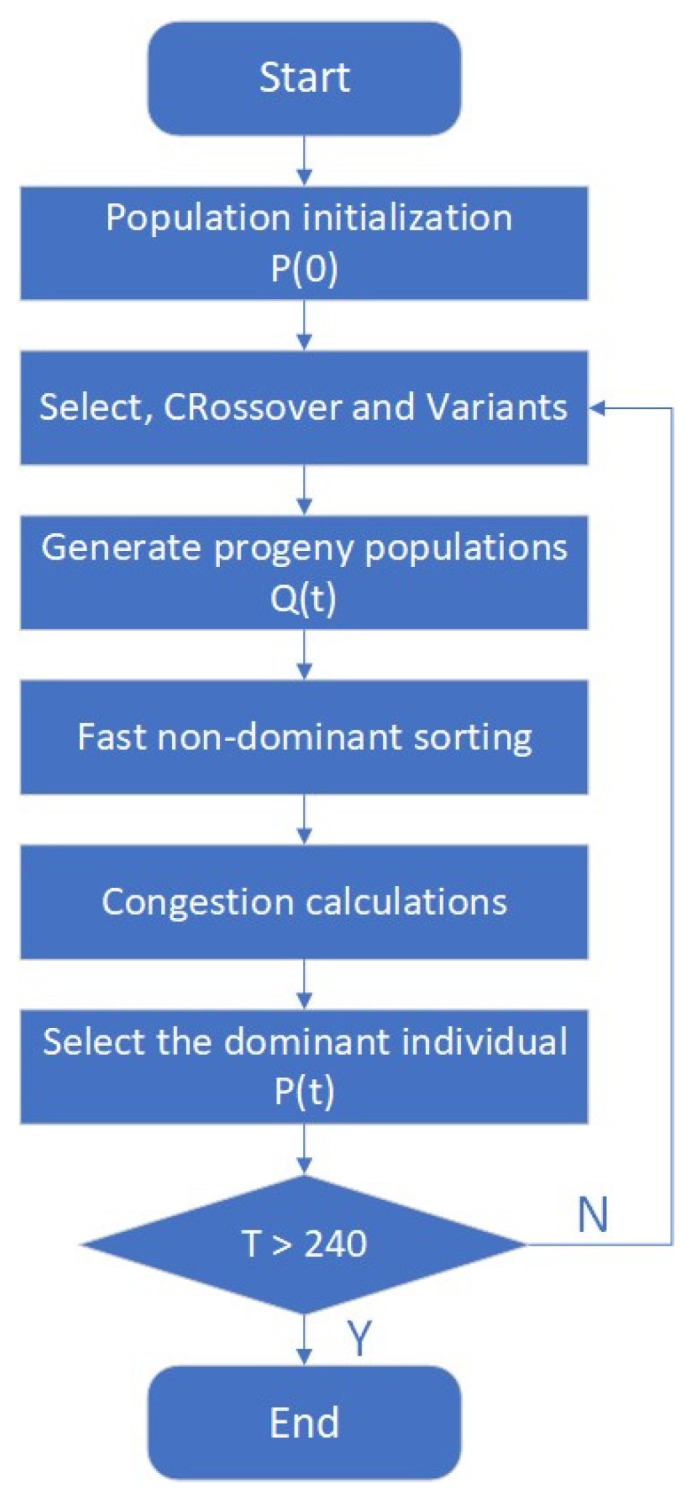
A flow chart of NSGA-II multi-objective optimization algorithm.

Once the configuration of the beams is established, some geometric parameters need to be optimized for increased structural efficiency. Hence, the NSGA-II algorithm was introduced to optimize the geometric parameters of the I-beams, with the objective function being to minimize mass, reduce stress, and maximize stiffness. The NSGA-II multi-objective optimization algorithm was adopted, in which each objective parameter is processed separately. The algorithm performs standard genetic operations of mutation and crossover on design variables. The selection procedure relies on two primary mechanisms: non-dominant sorting and crowding distance sorting, and its optimization flow chart is shown in Figure 5.

**Figure 6:**
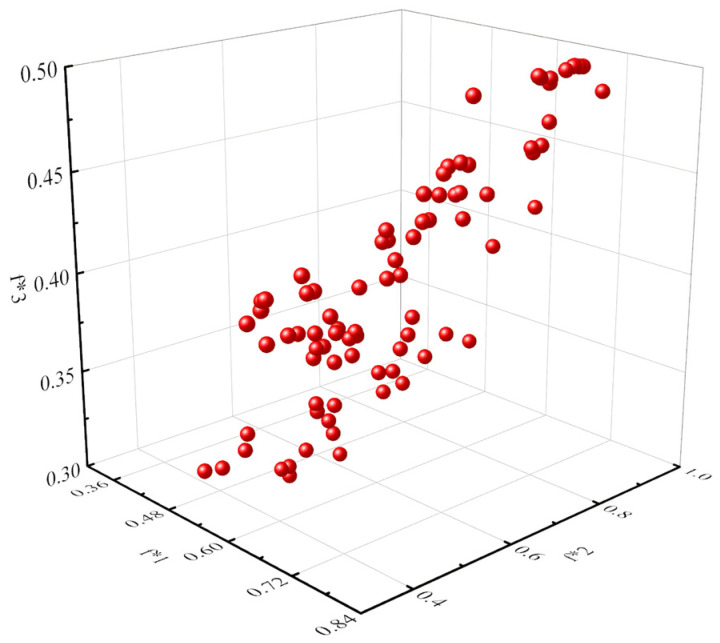
The collection of Pareto optimum solutions.

Multi-objective optimization aims to identify a collection of diverse solutions that together represent the optimal trade-off surface for several objectives. The surface generated in space is referred to as the Pareto front, specifically denoting the Pareto optimum solutions. Figure 6 displays the collection of Pareto optimum solutions that were obtained. And the following parameters were selected: *B*_1_ = 7, *B*_2_ = 25, *B*_3_ = 1.5, *H*_1_ = 2.1, *H*_2_ = 4.2, and *R* = 2.01. Due to the uneven tensile and compressive strength of 3D-printing materials, with the tensile strength being superior to the compressive strength, a thickness ratio of 1.5 was employed. The thickness of the upper and lower edge strips was determined according to the following formula:

(9) $$\begin{array}{c} \;H_{u}=H_{2}+(R_{ub}-1)\times H_{2}/(1+R_{ub})\\ H_{b}=H_{2}-(R_{ub}-1)\times H_{2}/(1+R_{ub}) \end{array}$$

here *H*_u_ is the thickness of the upper edge strip, *H*_b_ is the thickness of the lower edge strip, and *R*_ub_ is the ratio of the thickness of the upper and lower edge strips, which is 1.5.

### 2.5. Geometry Parameters of Configuration

**Figure 7:**
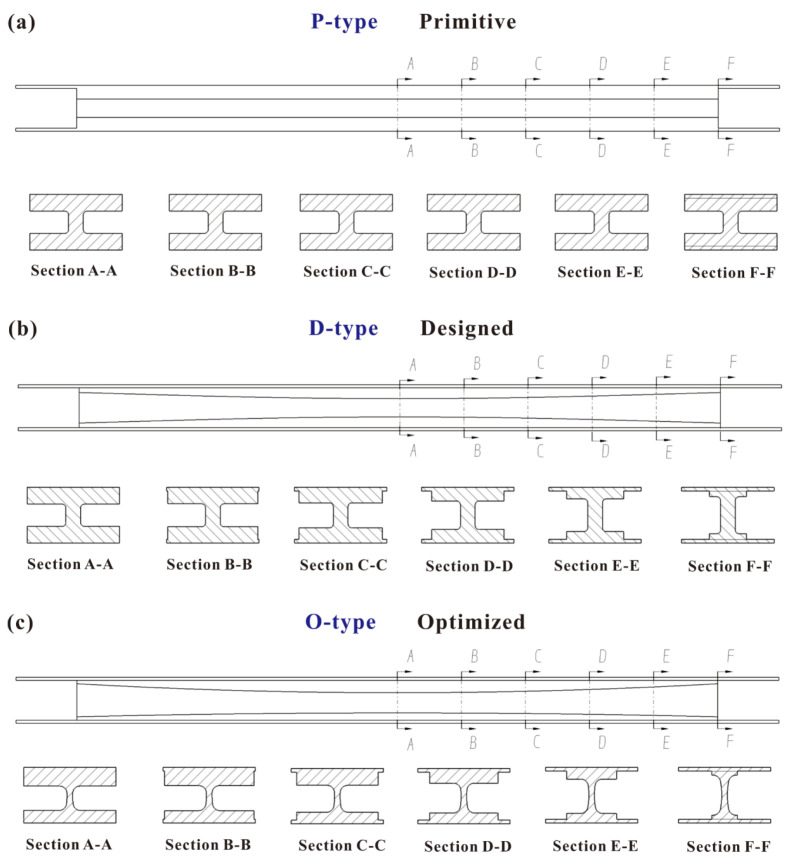
The geometry of variable cross-sections.

The geometry of variable cross-sections is presented in Figure 7, while the portions were divided into intervals of 21 mm and the sections were labeled sequentially from A-A to F-F. The corresponding parameters are listed in Table 3, in which *W*_b_ is the width of the beam, *W*_w_ is the width of the web, *H*_u_ is the thickness of the upper edge strip, and *H*_b_ is the thickness of the lower edge strip.

## 3. Results and Discussion

### 3.1. Flexural Properties of Different Configurations

**Figure 8:**
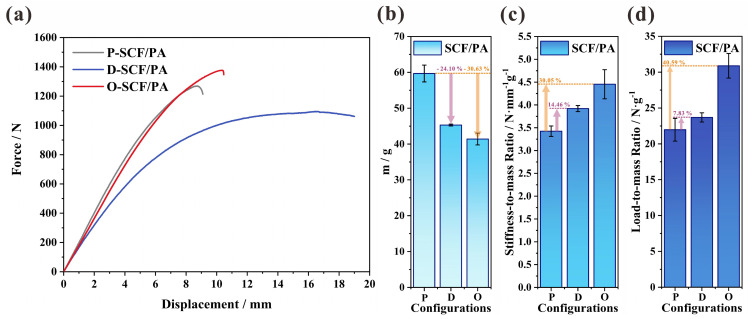
The flexural properties of the primitive, designed, and optimized configurations: (**a**) the force-to-displacement curve, (**b**) the mass, (**c**) the stiffness-to-mass ratio, and (**d**) the load-to-mass ratio.

Figure 8a illustrates the force-to-displacement curve of the primitive, designed, and optimized configurations of the SCFs-reinforced PAs. The P-type, D-type, and O-type I-beams exhibit linear characteristics at the beginning. Despite achieving a 30.63% reduction in mass through optimization (Figure 8b), the O-type I-beam still maintains a stiffness comparable to that of the P-type I-beam. However, the D-type I-beam demonstrates a significantly lower stiffness compared to both the O-type and P-type I-beams, while the mass is reduced by 14.46% compared to the P-type I-beams. Conversely, both the design and optimization resulted in an increase in the displacement of the 3D-printed I-beam at its maximum load as compared to the primitive one. This indicates that the 3D-printed I-beams’ ability to maintain structural integrity was improved. Furthermore, the enhancement of structural efficiency and the achievement of lightweight effects in the designed and optimized 3D-printed I-beams are prominently demonstrated in Figure 8c,d. Compared with the primitive one, the designed and optimized structures also exhibit a 14.46% and 30.05% increase in the stiffness-to-mass ratio and a 7.83% and 40.59% increase in the load-to-mass ratio, respectively. These increases mean that D-type and O-type I-beams have smaller deflections than P-type I-beams under identical load conditions. Moreover, when the design standard requires the same structural failure load, D-type and O-type I-beams have less weight compared to P-type I-beams.

**Figure 9:**
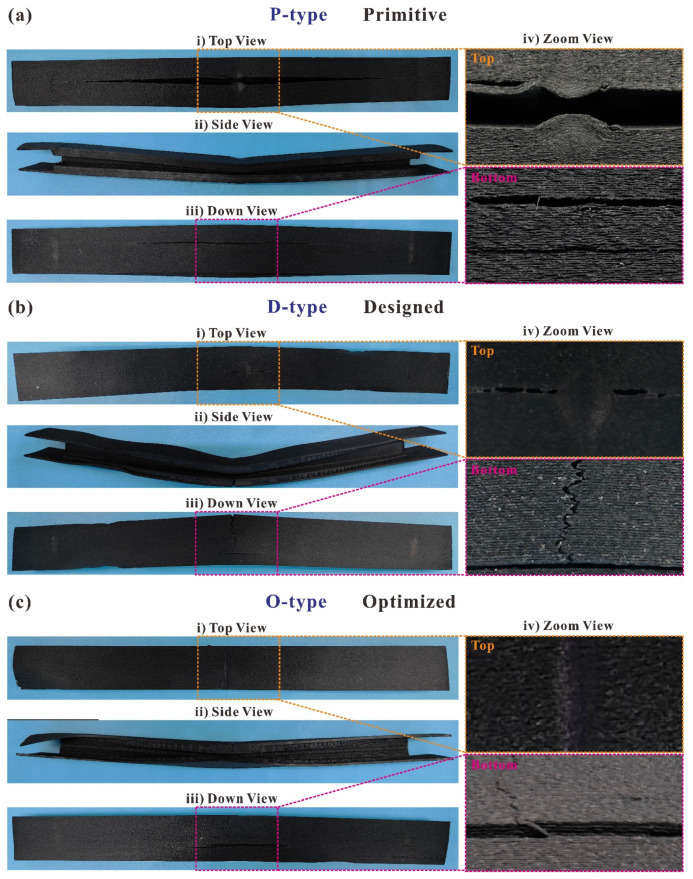
A comparison of the failed specimens of (**a**) the primitive, (**b**) designed, and (**c**) optimized configurations of SCFs-reinforced PAs in (**i**) top, (**ii**) side, (**iii**) down, and (**iv**) zoom views.

A comparison of the failed specimens of the primitive, designed, and optimized configurations of the SCFs-reinforced PAs is presented in Figure 9. In all three configurations of the 3D-printed I-beams, considerable residual plastic deformation was revealed after the three-point bending testing. Additionally, marginal circular cross-section indents remain as the result of the compression of the loading head. These indents are visible in the zoom view (iv) of the top surface in Figure 9a–c. The primary mode of failure for the P-type I-beam is delamination, where the separations between the 3D-printed layers propagate and extend dramatically along the length direction of the beams (Figure 9a). Furthermore, this delamination failure mode appears on both the top and bottom surfaces of the P-type I-beam. The occurrence of delamination leads to rapid structural failures, swift load drops, and the inadequate utilization of material properties. Because at this point, structural damage is dominated by weak interfaces between 3D-printed layers, which is an undesirable form of damage.

The predominant cause of failure for the D-type and O-type I-beams is a combination of delamination and tensile failure. The fractures caused by delamination failure exhibit elongated and narrow apertures, dispersed longitudinally over the top surface of the D-type I-beam, while the top surface of the O-type I-beam only has indents attributable to the loading head. The fracture, resulting from tensile failure, exhibits an irregular serrated shape that is perpendicular to the direction of length and is observed on the bottom surface of the D-type and O-type I-beams. This is consistent with the three-point bending conditions in which the top surface of the I-beams is subject to the tensile load, and the bottom surface is under compression. Improvements in failure modes impact the structural flexural properties of 3D-printed I-beams. As presented in Figure 9c, the appearance of tensile fractures and the disappearance of delamination fractures indicate that the properties of the material were utilized to a greater extent. This is further supported by the substantial enhancement of the O-type I-beam in comparison to its primitive configuration, specifically in terms of its structural flexural properties (Figure 8).

### 3.2. Flexural Properties of Different Reinforcements

**Figure 10:**
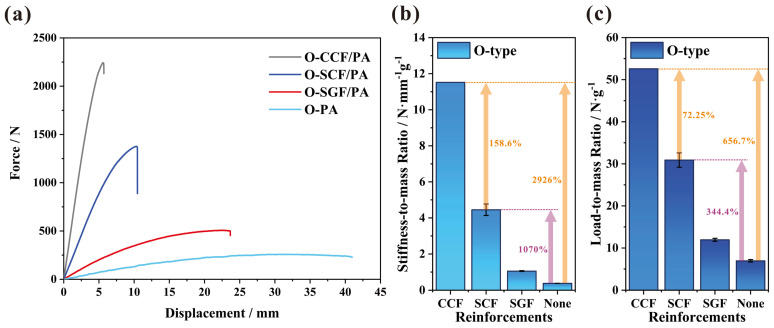
The flexural properties of CCFs-, SCFs-, and SGFs-reinforced PAs and PAs in the configuration of the O-type I-beam: (**a**) the force-to-displacement curve, (**b**) the stiffness-to-mass ratio, and (**c**) the load-to-mass ratio.

Figure 10a presents the force-to-displacement curve of the CCFs-, SCFs-, and SGFs-reinforced PAs and PAs in the configuration of the O-type I-beam. The 3D-printed I-beam of PAs with no fiber reinforcements demonstrates an extremely brief linear stage and exhibits obvious nonlinear characteristics in advance of attaining the peak load. However, fibers have the capacity to substantially reduce the nonlinearity exhibited by 3D-printed I-beams. Among them, the reduction effect of short carbon fibers has a more pronounced reduction impact compared to short glass fibers. Additionally, continuous carbon fibers have an even greater reduction effect than short carbon fibers. Nevertheless, the displacement of the peak load also decreases when fibers are added. This indicates that the addition of continuous fibers leads to a greater decrease compared to short fibers. Furthermore, the addition of short carbon fibers results in a greater decrease than short glass fibers. As the displacement at the peak load decreases, brittleness supersedes ductility as the prevailing property of the 3D-printed beams.

As shown in Figure 10b,c, due to the lower density of carbon fibers, the improvement in flexural properties is superior after taking into account the reduction in mass. Compared to the corresponding structure without reinforcements, the addition of CCFs increases the stiffness-to-mass ratio of the 3D-printed I-beam by 2926%. When the structure is subjected to the same design constraints for structural deformation, the improvement in the ratio of stiffness to mass allows for a substantial reduction in the weight of the structure. The continuous carbon fibers, which are evenly distributed along the length of the I-beam, serve as the framework for the beam structure. Working in conjunction with the matrix, they facilitate the smooth and uninterrupted transmission of loads. And the increase is 158.6% when compared to the corresponding structure with short carbon fibers. The addition of continuous carbon fibers greatly improves structural efficiency and significantly facilitates achieving lightweight structures. However, currently, printers and consumables for 3D printing with continuous carbon fibers are relatively expensive. Based on the supplier’s quotation, the price for 100 g of CCF/PA prepreg yarn is around USD 337.5, whereas SCF/PA consumables of equivalent quality are priced at only USD 5.5. Consequently, the utilization of short carbon fibers to enhance structures is a more cost-effective option in certain industrial application scenarios, such as the structural components of drones, battery packs of new energy vehicles, and medical device casings, etc. The addition of short carbon fibers increases the stiffness-to-mass ratio of the 3D-printed I-beams by 1070% in comparison to the I-beams 3D-printed by PAs, representing a highly notable improvement. Similarly, the addition of fibers also significantly improves the load-to-mass ratio, which indicates the load-bearing capacity per unit mass. Compared with the structure without reinforcements, continuous and short carbon fibers increase the load-to-mass ratio of 3D-printed I-beams by 656.7% and 344.4%, respectively.

**Figure 11:**
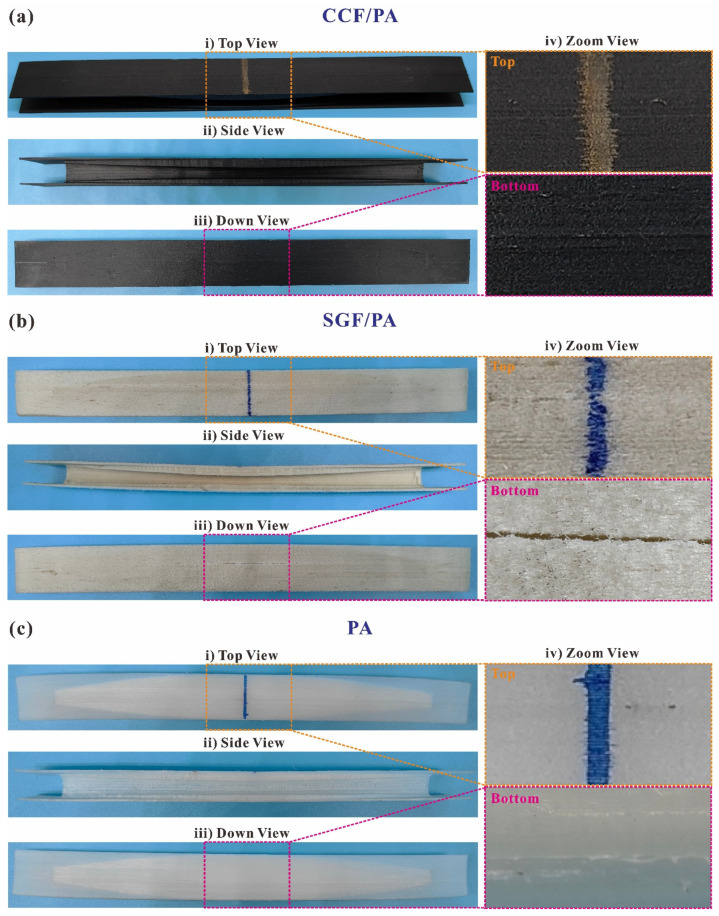
A comparison of the failed specimens of (**a**) CCFs-, (**b**) SGFs-, and (**c**) non-reinforced PAs in the configuration of the O-type I-beam in (**i**) top, (**ii**) side, (**iii**) down, and (**iv**) zoom views.

The comparison of the failed specimens of CCFs-, SGFs-, and non-reinforced PAs in the configuration of the O-type I-beam is presented in Figure 11. Both the CCFs- and non-reinforced 3D-printed I-beams exhibit little plastic deformation, while the underlying causes differ. The CCF-reinforced 3D-printed I-beam comprises over 50% of fiber content, while the content of the short glass fibers is 25% in the structures. Regarding the CCF-reinforced 3D-printed I-beam, it is important to note that the low resin content diminishes the structural plasticity. Conversely, the addition of CCFs leads to the structure attaining its maximum displacement at 5.61 mm and subsequently fracturing rapidly (Figure 10a). The limited structural displacement is insufficient to induce significant residual plastic deformation. For the 3D-printed I-beam made from PAs, the structural stiffness is relatively low (14.06 N·mm^−1^), resulting in a certain level of overall flexibility in the structure. Upon eliminating the loading boundary conditions, the entire structure rebounds, leading to negligible residual plastic deformation. As shown in Figure 11, considering the mode of failure, delamination is the primary factor affecting CCFs-, SGFs-, and non-reinforced 3D-printed I-beams. There is slight open cracking along the length direction on both their upper and bottom surfaces, and the cracking on the lower surface is relatively obvious.

### 3.3. Flexural Properties of Different Matrices

**Figure 12:**
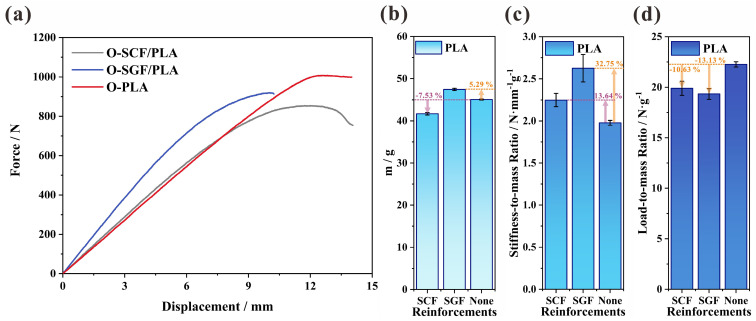
The flexural properties of SCFs- and SGFs-reinforced PLAs, and PLAs in the configuration of the O-type I-beam: (**a**) the force-to-displacement curve, (**b**) the mass, (**c**) the stiffness-to-mass ratio, and (**d**) the load-to-mass ratio.

Apart from the PA matrix, PLA is also utilized with SCFs, SGFs, and no reinforcements for 3D-printed O-type I-beams. And their flexural properties are illustrated in Figure 12. The force-to-displacement curve is present in Figure 12a, in which the addition of SCFs and SGFs both increase the stiffness of the structure to 96.69 and 124.55 N·mm^−1^, respectively. As depicted in Figure 12b, the mass of the O-type I-beams is reduced by 7.53% through the combination with SCFs. On the contrary, the mass of the structure is increased by 5.29% upon the addition of SGFs. Nevertheless, when stiffness and mass are both taken into account, SGFs prove to be a superior option. Both SGFs and SCFs contribute to an increase of 32.75% and 13.64% in the stiffness-to-mass ratio of the 3D-printed I-beams, respectively.

Nevertheless, the addition of short fibers results in a decrease in the load-to-mass ratio of the PLA I-beams. Both SGFs and SCFs contribute to a decrease of 10.63% and 13.13% in the stiffness-to-mass ratio, respectively (Figure 12d). As listed in Table 1, the PLA matrix has an elongation at break of 29.92%, which is much lower than the elongation of the PA matrix (196%). And the E-glass fiber has a 3% elongation at break, whereas the T300-carbon fiber only has 1.5% elongation at break. By incorporating short fibers, the elongation at break of the entire structure will decrease, particularly affecting PLA, which has an elongation at break that is less than one-sixth of PAs. The force-to-displacement curve also illustrates the substantial decrease in the peak load of SCF- and SGF-reinforced PLA I-beams in comparison to the structure with a pure matrix (Figure 12a).

**Figure 13:**
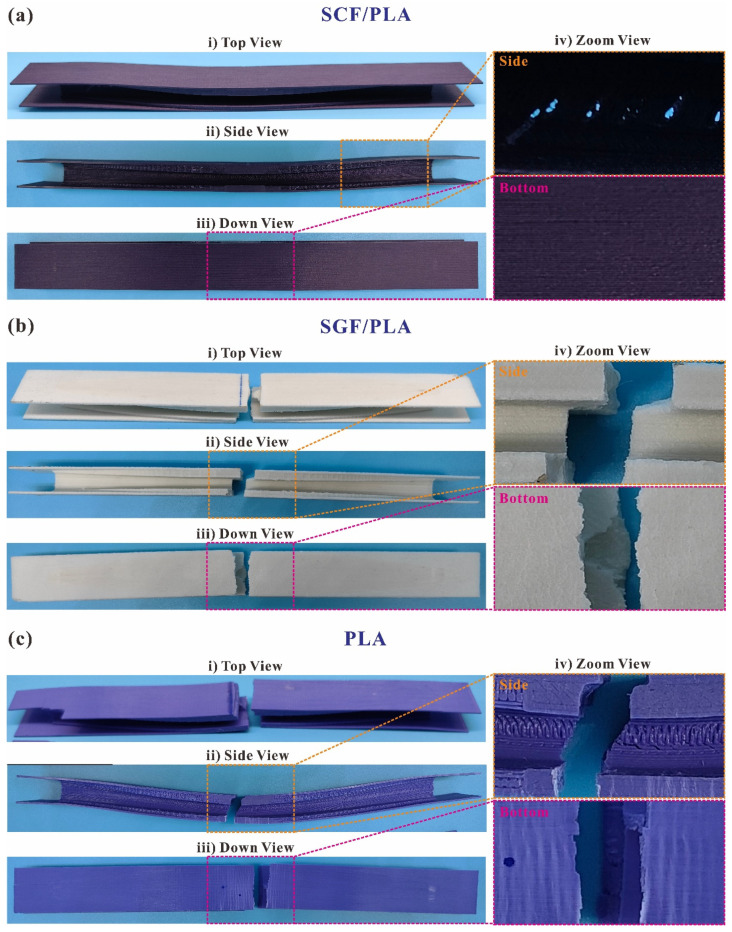
A comparison of the failed specimens of (**a**) SCFs-, (**b**) SGFs-, and (**c**) non-reinforced PLAs in the configuration of the O-type I-beam in (**i**) top, (**ii**) side, (**iii**) down, and (**iv**) zoom views.

The comparison of the failed specimens of SCFs, SGFs, and non-reinforced PLA in the configuration of the O-type I-beam is presented in Figure 13. Due to their comparatively high stiffness and load displacement at peak force, SGF-reinforced I-beams exhibit negligible residual plastic deformation, while the failed specimens of SCFs- and non-reinforced I-beams demonstrate substantial residual bending distortion. The SGFs- and non-reinforced I-beams exhibit extensive structural damage, accompanied by fractures with uneven and jagged edges that permeate the whole structure. During the experiment, initial observations revealed the presence of white creases on the surfaces, which subsequently progressed into the material’s fracture. The material’s fracture dominates, and the delamination cracking caused by 3D printing is not observed, which indicates that the materials were effectively utilized. Consequently, the SGFs- and non-reinforced I-beams demonstrate prominent peak loads (Figure 12a). The initial failure of the SCFs-reinforced I-beams primarily manifests on the web (Figure 13a), resulting in a reduction in load capacity, which is an undesirable mode of failure.

### 3.4. Material Selection

**Figure 14:**
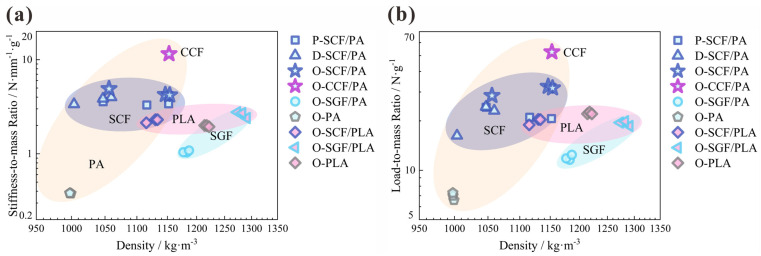
Ashby-type material selection maps: (**a**) stiffness-to-mass ratio and (**b**) load-to-mass ratio.

The experimental results of the indexes of flexural properties, including the stiffness-to-mass ratio and load-to-mass ratio, are summarized in the Ashby-type materials selection charts (Figure 14). The CCFs-reinforced PAs have the most remarkable structural lightweight effect compared to other materials tested in this study. This is noticeable in their significantly greater stiffness-to-mass ratio (11.52 N·mm^−1^g^−1^) and load-to-mass ratio (52.59 N·g^−1^). The SCFs-reinforced PAs have the potential to serve as economical substitutes for the CCFs-reinforced PAs. The 3D-prined I-beam of the O-type configuration produced by it has a relatively high stiffness-to-mass ratio (4.45 N·mm^−1^g^−1^) and load-to-mass ratio (30.89 N·g^−1^). The application of SGFs is not recommended, due to the lack of significant enhancement in mechanical properties and the subsequent increase in structure weight, unless it is employed in specific industrial scenarios where lightweighting is not required.

For the matrix of the 3D-printed composites, PAs and PLAs have extremely distinct properties. The 3D-printed I-beams with pure PA resin demonstrate the lowest bending characteristics among all specimens. They possess the average stiffness-to-mass ratio of 0.38 N·mm^−1^g^−1^ and the average load-to-mass ratio of 6.95 N·g^−1^. However, whether the addition of SGFs, SCFs, or CCFs indeed enhances the mechanical properties of the overall structure significantly remains to be determined. It is noted that reinforcements always play a crucial and beneficial function in PA composite I-beams. With regard to PLA resin, the mechanical performances are completely different. While the incorporation of SGFs and SCFs enhances the stiffness-to-mass ratio of the structure, it leads to premature structural failure and diminishes the load-to-mass ratio.

A comparison of the stiffness-to-mass ratio and load-to-mass ratio between this work and the literature is listed in Table 4. It should be noted that the flexural properties of beams are greatly affected by the span of supporting rollers. In general, the flexural properties tend to diminish as the span increases. Despite the fact that the span chosen for the present study is 200 mm, which is greater than the spans used in previous studies [9,26] (141 mm and 80 mm, respectively), the load-to-mass ratio demonstrates a reasonably similar level when identical material is used. As for the stiffness-to-mass ratio, this is more significantly influenced by the span than the load-to-mass ratio and becomes non-comparable across different spans. For example, the stiffness-to-mass ratio of [26] is only 4.53% of that of [9] when the span changes from 80 to 141 mm. Hence, it is suggested that the Ashby-type material selection maps should be used for the purpose of comparing the flexural properties of beams when the span and the design envelope (length, width, and height) are the same.

## 4. Conclusions

In this study, the design and optimization of I-beams were performed to obtain improved configurations. 3D printing was utilized to manufacture I-beams with three distinct configurations and seven kinds of materials. Three-point bending testing was conducted to verify the flexural properties of different configurations, reinforcements, and matrices. The following conclusions can be derived:

1. The flexural properties of 3D-printed I-beams were significantly improved via structural design and multi-objective optimization, which were based on the equal-strength philosophy and the NSGA-II algorithm, respectively. Design and optimization reduced the mass of I-beams made of SCFs-reinforced PAs by 24.10% and 30.63%, respectively. The structures also exhibited a 14.46% and 30.05% increase in the stiffness-to-mass ratio and a 7.83% and 40.59% increase in the load-to-mass ratio, respectively. The design and optimization method proved to be effective for 3D-printed I-beams.
2. Compared with pure PA structures, although the CCFs-reinforced PA I-beam exhibited a substantial improvement (2926%) in its stiffness-to-mass ratio, the addition of SCFs also increased the stiffness-to-mass ratio and the load-to-mass ratio by 1070% and 344.4%, respectively. Considering that the price of CCFs-reinforced PAs (USD 337.5) is much higher than SCFs-reinforced PAs (USD 5.5), SCFs-reinforced PAs have the potential to serve as an economical substitute for CCFs-reinforced PAs. The 3D-prined I-beam of the O-type configuration produced by SCF-reinforced PAs had a relatively high stiffness-to-mass ratio (4.45 N·mm^−1^g^−1^) and load-to-mass ratio (30.89 N·g^−1^), while the corresponding PA structure had a stiffness-to-mass ratio of 0.38 N·mm^−1^g^−1^ and a load-to-mass ratio of 6.95 N·g^−1^.
3. Compared with PLAs (elongation at break of 29.92%), PAs (elongation at break of 196%) are a better choice of matrix to demonstrate the adverse effects of reinforcements. The addition of reinforcements, such as SGFs, SCFs, or CCFs, improves the mechanical properties of PA composite I-beams to a great extent. However, in PLA resin, the incorporation of SGFs and SCFs increased the stiffness-to-mass ratio but caused premature structural failure and reduced the load-to-mass ratio. Although the addition of SCFs increased the stiffness-to-mass ratio of PLA I-beams by 13.64%, the load-to-mass ratio was reduced by 10.63%.

This work provides valuable insights into the design and optimization of 3D-printed I-beams, specifically focusing on their flexural properties and material selection. Optimization using the NSGA-II algorithm and structural design based on the equal-strength philosophy were both demonstrated to be effective for 3D-printed I-beams. Simultaneously, it is worth noting that the mechanical performances of 3D-printed materials are diminished during compression as opposed to tension. When financial considerations permit, CCFs are the optimal material for enhancing mechanical properties. SCFs, however, are comparatively economical alternatives. It was discovered that the highly ductile resin matrix served as a successful medium for adding reinforcements. These findings could facilitate the lightweight design and optimization of bending-resistant structures.

## Figures and Tables

**Table 1:** The type and mechanical properties of printing materials.

Suppliers	Type	Tensile Strength/MPa	Modulus/GPa	Elongation at Break/%	Density/g·cm^−3^
Markforged	PA-CCF	800	51	1.5	1.4
	Onyx	35.71	3.59	4	1.2
eSUN	ePAHT-CF	173.37	5.61	8.93	1.4
	ePA-GF	76.93	1.72	20.07	1.35
	ePA	57	1.5	196	1.12
	ePLA-CF	28	3.55	4.27	1.21
	ePLA-GF	59.27	4.41	7.99	1.31
	ePLA-Lite	61.34	3.82	29.92	1.23

**Table 2:** Parameters of 3D printing.

Type	Layer Thickness/mm	Nozzle Temperature/°C	Hatch Space/mm	Printing Speed/mm·s^−1^
PA-CCF	0.125	275	1	15
Onyx	0.2	260	0.5	15
ePAHT-CF	0.2	240	0.5	50
ePA-GF	0.2	240	0.5	50
ePA	0.2	240	0.5	50
ePLA-CF	0.2	210	0.5	50
ePLA-GF	0.2	210	0.5	50
ePLA-Lite	0.2	210	0.5	50

**Table 3:** Geometry parameters of configuration.

Type	Section Lable	*W*_b_/mm	*W*_w_/mm	*H*_u_/mm	*H*_b_/mm
P	A-A	25	4	4.5	4.5
	B-B	25	4	4.5	4.5
	C-C	25	4	4.5	4.5
	D-D	25	4	4.5	4.5
	E-E	25	4	4.5	4.5
	F-F	25	4	4.5	4.5
D	A-A	25	4	4.5	4.5
	B-B	24.4	4	4.41	4.41
	C-C	22.6	4	4.12	4.12
	D-D	19.6	4	3.66	3.66
	E-E	15.4	4	3.09	3.09
	F-F	10	4	2.5	2.5
O	A-A	25	1.5	5.04	3.36
	B-B	24.28	1.5	4.92	3.31
	C-C	22.12	1.5	4.57	3.16
	D-D	18.52	1.5	3.98	2.91
	E-E	13.48	1.5	3.16	2.55
	F-F	7	1.5	2.1	2.1

**Table 4:** Comparison between this work and the literature.

Configuration	Materials	Span/mm	H/mm	Stiffness-to-Mass Ratio/mm	Load-to-Mass Ratio/mm	Reference
O-type	CCF/PA	200	15	11.52	52.59	This work
O-type	SCF/PA	200	15	4.45	30.89	
D-type	SCF/PA	200	15	3.92	23.69	
Corrugated core	CCF/PA	141	23.2	28.49	57.98	[26]
	SCF/PA	141	23.2	10.6	23.44	
Diamond core	CCF/PA	80	10	627.7	68.33	[9]

## Data Availability

Data can be obtained from the authors on request.
